# Predictive Value of Hypomineralization of Second Primary Molars for Molar Incisor Hypomineralization and Other Relationships between Both Developmental Defects of Dental Enamel

**DOI:** 10.3390/jcm12175533

**Published:** 2023-08-25

**Authors:** María Marcianes, Pablo García-Camba, Alberto Albaladejo, Margarita Varela Morales

**Affiliations:** 1 Unit of Orthodontics, University Hospital Fundación Jiménez Diaz, 28040 Madrid, Spain; 2 Department of Surgery, Faculty of Medicine, University of Salamanca, 37008 Salamanca, Spain

**Keywords:** child, dental enamel, enamel opacities, incisor, molar, molar hypomineralization

## Abstract

Molar incisor hypomineralization (MIH) and hypomineralized second primary molars (HSPM) are dental enamel developmental defects with significant prevalence and psychophysical impact, posing a public health concern. We examined the relationship between HSPM and MIH in 426 children aged 7 to 10 years (213 with MIH, 213 without MIH). The frequency of HSPM was higher in children with MIH (37.1% vs. 11.3%, *p* < 0.001). However, there was no correlation between MIH severity and presence of HSPM (*p* = 0.296). In patients with both defects (n = 79), 90% of severe MIH cases were associated with severe HSPM, while 87.8% of mild MIH cases were associated with mild HSPM (*p* < 0.001). The involvement of permanent incisors by MIH was not influenced by the presence of HSPM or HSPM severity. HSPM exhibited a positive predictive value of 76.7, negative predictive value of 58.8, sensitivity of 37.1% (95% CI = 30.6–43.6%), and specificity of 88.7% (95% CI = 84.5–93%). The odds ratio for MIH based on HSPM was 4.64 (95% CI). HSPM may serve as a predictor of MIH, but its absence does not rule out the occurrence of MIH. The results highlight the need for surveillance programs in children presenting with HSPM to facilitate early diagnosis and preventive measures for MIH-related complications.

## 1. Introduction

Molar incisor hypomineralization (MIH) and hypomineralization of second primary molars (HSPM) are qualitative developmental defects of dental enamel that, because of their high worldwide prevalence and potential negative impact on patient health and quality of life, should be considered a public health problem [1]. HSPM and MIH share many clinical, physiopathological and structural features [2]. In HSPM, the defect involves between one and four second primary molars (SPM), while in MIH the affected teeth are one or more of the first permanent molars (FPM) and, in a variable proportion of cases, the permanent incisors [3]. Though a less common finding, other permanent teeth may show lesions of hypomineralization [4]. HSPM and MIH lesions are macro- and microscopically similar. Macroscopically, they vary in size and severity, ranging from small, well-demarcated white, yellow, or brown asymmetric opacities to extensive lesions secondary to post-eruptive breakdown (PEB) and caries. Wide inter-rod spaces and changes in the mineral concentration of Ca, P and C are the main microstructural findings [5,6]. When hypomineralization is severe, particularly when associated with secondary PEB and caries, the lesions can produce intense hypersensitivity and need repeated treatment, and even the extraction of the affected teeth [7]. In such cases, the impact on the patient’s quality of life can be substantial, particularly when aesthetics are compromised by significant discoloration and breakdown of the incisors [8,9].

MIH and HSPM are both attributed to a disturbance during the prenatal, perinatal and early postnatal process of initial calcification and/or maturation of the enamel of the affected dental germs [10,11]. Although the number of studies addressing the possible risk are yet to be fully defined, it appears to be multifactorial, with the involvement of several systemic genetic and/or epigenetic factors acting synergistically or additively [12,13].

In a narrative review conducted by Butera et al. [14] in 2021, which analyzed twenty-five articles focusing on genetic, pre-, peri-, and postnatal risk factors associated with HSPM and MIH, several postnatal factors were found to have statistical significance. Among the postnatal factors identified, the review highlighted the relevance of the breastfeeding period, asthma, high fever episodes, infections/illnesses, chickenpox, antibiotic intake, diarrhea, and pneumonia in relation to HSPM and MIH.

However, in a later published study involving a population of 893 school children aged 8 to 10 years in Saudi Arabia, neonatal jaundice was the only etiological factor that showed a significant association with this disorder [15].

The reported global prevalence of these disorders is highly variable and seems to be increasing in recent years [16], perhaps owing to more precise recognition. For MIH, most studies have reported prevalences between 2.8 and 27.7% in different countries, with a global average of 14% [17,18]. However, some authors have reported prevalences approaching 40% [15]. Regarding HSPM, a meta-analysis showed an average worldwide prevalence of 6.8% on a child level and 4.1% on a tooth level [19]. This variability may be due in part to the absence of uniform criteria when defining cases and other methodological discrepancies between published epidemiological studies, which have created confusion and have hindered the output of systematic reviews and meta-analyses [20].

Diagnostic criteria for MIH were finally agreed upon by the European Association of Pediatric Dentistry (EAPD) in 2003 [21]. These criteria were later revised and updated in 2010 [22] and adapted for HSPM [23]. Even after their release and the proposal of a standard protocol for clinical field surveys [24], however, some studies continue to be published with heterogeneous and unclear criteria.

The association between HSPM and MIH in a single patient is not uncommon, and it has been suggested that since SPM erupt some years before FPM, HSPM could be a predictor of MIH. This possible relationship is highly important in pediatric dentistry, since the diagnosis of HSPM as soon as SPM erupt makes it possible to initiate more intensive supervision and preventive measures, as these patients are otherwise prone to developing MIH in permanent teeth within a few years. The first studies aimed at evaluating this hypothesis reported contradictory results [10,25,26,27,28,29], although a subsequent systematic review supported the predictive value of HSPM for MIH [30]. However, the authors of this review emphasized the heterogeneity and methodological limitations of some of the studies, and subsequent research has made little progress toward resolving the controversy [31]. In the present study, measures have been taken to control for methodological shortcomings to the greatest degree possible as concerns not only the diagnostic criteria for MIH and HSPM, but also the size of the samples, training and calibration of evaluators, and other aspects.

The purpose of this cross-sectional study was to investigate possible relationships between HSPM and MIH. The specific objectives were as follows: to assess the predictive value of HSPM for the development of MIH, to compare the frequency of HSPM between two groups of children, one with MIH and one without, to investigate a potential correlation between the severity of HSPM and the severity of MIH in a group of children with both disorders, and to explore the association between the severity of HSPM and the involvement of incisors in MIH within the same patient population. The null hypotheses tested in this study were as follows: HSPM does not serve as a predictive factor for the development of MIH in permanent teeth; there is no significant difference in the frequency of HSPM between children with and without MIH; and in children with both disorders there is no correlation between the severity of HSPM and the severity of MIH or the involvement of incisors by MIH.

## 2. Materials and Methods

### 2.1. Study Design

This cross-sectional study was part of a wider research project on the possible associations of MIH with other disorders, including HSPM, and anomalies of number, size, or eruption of teeth, conducted in the Orthodontics Unit of University Hospital Fundación Jiménez Díaz (UHFJD) in Madrid, Spain.

### 2.2. Sample Size and Procedure

A sample of 426 children (213 patients with MIH and 213 age-matched patients without MIH) whose clinical records were most recently entered into the database of the Orthodontics Unit of UHFJD was selected. The age of the children was 7 to 10 years when their first diagnostic records were obtained. The frequency of MIH in the overall study population was 12.8%.

The selection of patients for the MIH sample began with the last patient included in the database who met the EAPD diagnostic criteria for MIH [21,22,23] (Table 1) following the application of the inclusion/exclusion criteria as described in Table 2. Subsequent patient recruitment proceeded in reverse order. The comparison sample of subjects without MIH was assembled in the same way.

### 2.3. Training and Calibration of the Examiners

The selection of MIH and non-MIH patients and the identification of those with HSPM were performed by 2 examiners trained and calibrated by a senior researcher. For training and calibration, we used a set of 50 photographs depicting MIH and HSPM of variable degrees of severity together with other enamel defects such as fluorosis, hypoplasia, and other opacities and discolorations. The Cohen’s Kappa inter-examiner coefficient was rated as outstanding (0.85). Intra-examiner reliability tests revealed near-perfect agreement. The senior researcher, considered the gold standard, discussed possible discrepancies with the 2 examiners, and questionable cases were not included in the samples.

### 2.4. Evaluation of Hypomineralization Lesions

MIH and HSPM lesions were assessed by means of high-quality digital intraoral photographs projected onto a 40-inch screen [32]. In accordance with the EAPD criteria, MIH was diagnosed when at least one FPM presented hypomineralization. The remaining FPM could be free of MIH or even absent. Therefore, for a subject to be included in the MIH sample, it was not necessary to have all FPMs present when at least one was affected by MIH. Lesions on the incisors were only diagnosed as MIH if at least one FPM was also involved. For the purposes of this study, it was agreed that the largest MIH opacity must have a diameter of at least 1 mm, according to the Developmental Defects of Enamel (DDE) index [33]. To avoid false-negative results for MIH, patients included in the non-MIH sample were required to have all 4 FPM present and free of MIH.

HSPM was diagnosed according to the adapted EAPD criteria for MIH [23]. A single affected SPM was sufficient for a subject to be considered HSPM-positive, but HSPM-negative status was only assigned if all SPM were free of the defect. As in the case of MIH lesions, the largest opacity had a minimum diameter of at least 1 mm in all cases.

### 2.5. Severity of MIH and HSPM

Severity ratings for MIH and HSPM were based on the lesion with the greatest severity. White, yellow-brown, or even dark brown delimited opacities, regardless of their size, were counted as mild MIH (or HSPM). PEB associated with opacities, atypical extensive caries with surrounding opacities, or atypical restorations, crowns, or extractions due to MIH (or HSPM) were criteria for severe MIH (or HSPM) [34]. In the MIH sample, any involvement of incisors in addition to the FPM was recorded.

### 2.6. Statistical Analysis

Mean and standard deviation were used to summarize quantitative variables, and frequencies and percentages for qualitative variables. Relations between qualitative variables were studied using contingency tables and Chi-squared test, or Fisher’s exact test as required.

Comparisons between groups regarding quantitative variables were performed using Student’s *t* test. The frequency of HSPM was calculated in both samples of patients with and without MIH and compared using Chi-squared test.

The degree of severity of MIH and the frequency of involvement of permanent incisors and other teeth were related to the severity of previous HSPM by means of a Chi-squared test.

To assess the predictive value of HSPM for MIH, a sensitivity and specificity analysis was performed, and positive and negative predictive values and odds ratio were calculated. The level of significance was set at *p* < 0.05.

### 2.7. Ethical Approval

The clinical study was approved by the hospital ethics committee for research in humans, under procedure number EO131-19_FJD. All methods were in accordance with approved guidelines.

## 3. Results

### 3.1. Descriptive Statistics

The MIH and non-MIH samples were age-matched. The mean ages of subjects were 8.43 ± 0.93 and 8.46 ± 0.92, respectively. Table 3 shows the descriptive statistics of the study population, including the sex, age and age distribution of subjects with and without MIH, including the respective number of children aged 8, 9, 10, and 11 years in each sample.

Comparisons with respect to age were not statistically significant, which was to be expected given that the selection of the groups was age-matched. There were also no significant differences in sex and age group distribution, aspects that were not considered in the sample selection method.

### 3.2. HSPM as a Predictor for MIH

Table 4 shows the data for HSPM as a predictor for MIH. Analyses showed a sensitivity of 37.1% (95% CI = 30.6–43.6%), indicating that somewhat more than one third of the patients with MIH had previously developed HSPM. The specificity was 88.7% (95% CI = 84.5–93%), indicating that about 90 percent of the children who did not have MIH also did not have previous HSPM. The predictive positive value was 76.7 and the negative predictive value was 58.8. That means that 76.7% of the patients who showed HSPM had developed MIH, but also 58.8% of the patients who did not have HSPM had developed MIH. The absence of HSPM in primary dentition does not rule out the subsequent development of MIH. The odds ratio was 4.64 (95% CI = 2.79–7.71), which indicated that children with HSPM were 4.64 times more likely to present MIH than those who did not develop HSPM.

### 3.3. Frequencies of HSPM in Patients with MIH and without MIH

The frequencies of HSPM in patients with MIH and without MIH were 37.1% (79 from 213 patients) and 11.3% (24 from 213 patients), respectively (*p* < 0.001) (Table 5).

That is, patients with MIH showed HSPM significantly more frequently than those without MIH.

### 3.4. Relationship between Severity of MIH and the Presence of HSPM

No association was found between the severity of MIH and the presence of HSPM (*p* = 0.296) (Table 6).

That is, patients who presented severe MIH were not more likely to have HSPM than those patients with mild MIH. However, a slightly higher percentage of patients with HSPM was observed in the severe MIH group compared to the mild MIH group. This observation could suggest a trend, and a much larger sample would be necessary to confirm it.

### 3.5. Relationship between MIH Severity and the Severity of HSPM

Table 7 shows the relationship between the severity of HSPM and the severity of MIH in the 79 subjects with a co-occurrence of both defects. Mild disease was the most common form observed in both MIH and HSPM; 90% of cases of severe HSPM were associated with severe MIH, and only 12.2% of cases of severe HSPM had developed mild MIH (*p* < 001).

### 3.6. Relationship between HSPM Severity and the Involvement of Only Molars or Molars plus Incisors by MIH

In our sample of 213 patients with MIH, we observed a slight predominance of the inciso-molar form over the only molars form (58.7% vs. 41.3%), although this difference did not reach statistical significance. This predominance was observed irrespective of whether the patients with MIH had HSPM or not. We also found that in patients with MIH who presented HSPM (n = 79) there was no significant relationship between the severity of HSPM and the frequency of inciso-molar or molar forms of MIH (Table 8).

## 4. Discussion

In the last decade, clinicians and researchers have become increasingly aware of the importance of developmental defects in dental enamel at the individual and community level [1]. However, most studies have focused on the epidemiological and clinical aspects of MIH and less attention has been paid to HSPM or the relationship between HSPM and MIH. A recent bibliometric analysis of worldwide trends in published research on MIH and HSPM over the last two decades showed an increase in scientific publications, especially in more recent years, though a majority of studies (87.4%) were on MIH, while only 4.6% explored the link between HSPM and MIH [35]. Furthermore, the methodological inconsistencies and other limitations that have hampered some studies on MIH are even more commonplace in research on HSPM, which has limited the validity of their conclusions [20]. This gap in the knowledge base may come as a surprise given the importance of considering HSPM as a possible predictor of MIH [30].

In this study, which analyzed the predictive value of HSPM for the subsequent development of MIH and other relationships between HSPM and MIH, we aimed to control for some of the aforementioned factors to the greatest extent possible. These measures include the use of standardized diagnostic criteria for MIH and HSPM, observer training, and careful selection of the study design, including subject age range, sample size, and inclusion/exclusion criteria, among other factors. However, other shortcomings were unavoidable, as discussed below.

### 4.1. Type of Population

The subjects of this research were patients who presented to an orthodontics clinic. This characteristic could have introduced some form of bias in prevalence studies if the presence of MIH influenced the demand for orthodontic treatment. However, this was not a study of prevalence. Furthermore, both samples were drawn from the same orthodontic population by applying the same inclusion/exclusion criteria, thus ruling out this possible bias. Moreover, the frequency of MIH in the UHFJD orthodontic database for the age range spanning 7 to 10 years (around 12%) was similar to or even lower than that appearing in epidemiological reports published on school or general populations [17,18,29,36]. Therefore, with respect to the frequency of MIH, the orthodontic sample used for the present study can be considered comparable to a sample of schoolchildren of the same age range. This finding is in line with the results reported by Landford et al. [37], who found a prevalence of MIH in a sample of orthodontic patients that was consistent with the previously reported worldwide prevalence among the general population. The fact that this sample was obtained from the orthodontics unit of a general hospital could also constitute a bias, though the unit of the UHFJD is a standard office, and as such does not treat a greater proportion of patients presenting syndromes or other diseases, otherwise excluded as per the inclusion/exclusion criteria. Other published research such as the studies by Elfrink et al. [10] or Negre-Barber et al. [29] has also been conducted in odontopediatric units located in medical centers or universities.

### 4.2. Patient Age

The age range of subjects has also hindered standardization in research on MIH and HSPM frequencies and relationships. In the present study, all children were between 7 and 10 years of age, which allowed for the coexistence of FPM and SPM according to the research objectives and design. In the studies of Ghanim et al. [25] and Estivals et al. [36], children were between 7 and 9 years of age, and in the study of Negre-Barber et al. [29] they were between 8 and 9. The age range in the research of Da Silva et al. [27] was from 6 to 11 years. It has been pointed out that the optimal age for diagnosing HSPM is around 5 years, when the SPM are fully erupted in most children. However, experts recommend the diagnosis and evaluation of MIH at age 8 years, when the FPM and permanent incisors should be present [30]. Premature evaluation (age 5 or 6 years), while somewhat better for assessing HSPM, could overlook some MIH-positive subjects due to the incomplete eruption of FPM. The age of 7 years could also be considered too early to assess MIH; however, as a single involved molar is sufficient to diagnose this defect [21,22,23], 7-year-old children with at least one molar showing MIH were included in the MIH sample of the present study if they met the other inclusion criteria, even if the remaining teeth had not erupted. One such criterion was the retention of all SPM (unless one presented HSPM), which is highly probable at 7 years of age. Extending the upper limit beyond 10 years of age could also lead to diagnostic errors, as lesions due to wear, caries, or other enamel defects could mask the hypomineralization lesions, and affected molars may have been extracted without any record of the motive.

### 4.3. Study Design and Sample Sizes

The present study was cross-sectional. To assess the predictive value of HSPM for MIH, a longitudinal design would be optimal, performing two examinations at ages 5 and 8 years to monitor dental development from the complete eruption of the SPM until the eruption of the FPM. However, this protracted longitudinal design poses logistical problems, particularly in younger children, and requires very large sample sizes due to the high number of patients lost to follow-up [30]. To our knowledge, the research by Costa-Silva et al. [26] is one of the few existing longitudinal studies assessing the predictive value of HSPM for MIH; most other published reports are cross-sectional [10,27,28,29]. The prospective study by Noor Mohamed et al. [38], which was conducted in a cohort of 287 preterm low birth weight (PT-LBW) children and 290 control full-term normal birth weight subjects followed up for 3 years, was not focused on the predictive character of HSPM for MIH. However, it showed a significant association between PT-LBW, the frequency of HSPM, and the incidence of MIH.

Regarding sample size, the relatively low worldwide prevalence of both defects (14% for MIH and around 6% for HSPM) and despite the large populations of children examined in most of the published studies, the sample sizes of patients with a co-occurrence of MIH and HSPM tend to be small. This fact was mentioned as a limitation in the systematic review by Garot et al. [30].

The design of the present study differs from other reports in that it was based on two independent pre-selected samples of 213 children each, one with MIH and one without. More than 3000 charts were reviewed to select these samples as a starting point for this study. One strength of our design is that the group of patients with a co-occurrence of MIH and HSPM (n = 79) was large enough to permit reliable calculations. In the sample of Mittal and Sharma [28], only 18 of 978 schoolchildren showed both defects, in the sample of Ghanim et al. [25] 21 of 809, and in the research of Negre-Barber et al. [29], 11.1% of 414 children.

#### 4.3.1. Diagnostic Criteria for HSPM

The adapted criteria proposed by the EAPD for MIH in the permanent dentition [21,22] were used to characterize HSPM [22]. Since the EAPD criteria were published, they have been widely adopted in epidemiological and clinical studies on MIH, but much less so in research on HSPM [4,10,27,28,36] which has been noted as one of the most important shortcomings of research on the subject [25].

#### 4.3.2. Characterization of the Lesions

In the present study, when characterizing hypomineralization lesions of MIH and HSPM, the largest opacity was required to have a diameter of at least 1 mm according to the DDE index of the Federation Dentaire Internationale (FDI). This criterion has been used in some studies [26,29], while others have used a minimum diameter of 2 mm [25,28] or no size limit at all [10]. These researcher-determined differences influence prevalence figures. Here, this possible bias is unlikely to have had an effect, as the frequency of HSPM was compared in two independent samples applying the same size criteria to qualify a lesion.

Although most epidemiological research on enamel developmental defects in schoolchildren or the general population have been performed by direct clinical examination [25,26,28,36], this study, like others [10], was based on high-resolution digital photographs. Such images have been used to detect and characterize enamel lesions with higher validity and reproducibility than direct clinical examination, which is highly influenced by examination conditions [32,34].

### 4.4. Relationships between HSPM and MIH

The results of the present study showed that the occurrence of HSPM was associated with the development of MIH in a sample of children, and that prior to permanent tooth eruption, HSPM may be predictive for MIH. These findings, based solely in the presence/absence of the defects, are in line with those of Mittal and Sharma [28], Negre-Barber et al. [29] and Estivals et al. [36] and with the conclusions of the meta-analysis by Garot et al. [30], but they contrast with those reported by Ghanim et al. [25], Elfrinck et al. [10] and Sidhu et al. [31].

The positive and negative predictive values in the present study indicated that although children with HSPM are more likely to subsequently develop MIH, the absence of the defect in the primary dentition does not rule out the appearance of MIH in the permanent molars. These results are consistent with those of some authors, but not all. Mittal and Sharma [28] examined 978 schoolchildren aged 6 to 8 years in India and found that 55 presented HSPM, 72 MIH, and 18 a co-occurrence of both defects. The authors concluded that the presence of HSPM was associated with a greater probability of MIH development in the future. Negre-Barber et al. [29] drew the same conclusion from their study of a sample of 414 Spanish children aged 8 and 9 years, 24.2% with MIH, 14.5% with HSPM and 11.1% with co-occurrence of the two defects. Da Silva et al. [27], in a sample of Brazilian children, found that MIH was six times more likely to occur in children with either HSPM or hypomineralized primary canines. Estivals et al. [36] in a study conducted in France found that a child with HSPM was more likely to have MIH than a child without HSPM, with an OR of 6.0. In contrast, Sidhu et al. [31], in a Canadian sample of 429 children with MIH and HSPM prevalence rates of 12.4% and 5.2%, respectively, found that hypomineralized SPM were not predictive for MIH. In a study conducted on a cluster sample of 809 Iraqi schoolchildren aged 7–9 years, Ghanim et al. [25] found that 53 had HSPM and 21 a combination of MIH and HSPM. Although this association did not reach statistical significance, the authors attributed their results to the small sample size and older age of the patients studied. Costa-Silva et al. [26] also found no association, and the sample size was similarly small.

### 4.5. Severity of MIH and HSPM

In the present study, most cases of MIH and HSPM were mild, as reported by some authors [2,25,28,29] but contrasting with others [10]. In cases of a co-occurrence of HSPM and MIH, no relationship was found between the occurrence of HSPM and the severity of MIH or between the severity of HSPM and the frequency of involvement of permanent incisors by MIH. However, a significant relationship was found between the severity of previous HSPM and subsequent MIH severity, such that if the HSPM was severe, MIH was more likely to be severe; if it was mild, MIH tended to be mild.

Other studies have analyzed various aspects of the relationship between the severity of HSPM and MIH and their respective frequencies, with varying results [25,28,29]. Among these authors, Mittal et al. [28] found a higher prevalence of MIH in the presence of mild HSPM. Taking a different approach, Elfrink et al. [10] reported a higher prevalence of severe HSPM in patients with MIH. The discrepancies between results could be due to the proportions of older and younger children in the samples analyzed. The severity of MIH/HSPM in a subject is not a constant parameter, but may increase over time, as teeth that remain longer in the mouth are more likely to develop PEB with or without caries [26], a complication that determines the characterization of an MIH or HSPM lesion as severe following the criteria used in the present study and elsewhere [27]. Therefore, a higher proportion of severe cases would be expected in subjects aged 9 and 10 years compared to those aged 7 and 8 years. This circumstance makes it difficult to compare the results of studies evaluating patients of different age ranges. Other authors have proposed lesion color as a criterion of severity, with severity increasing from white to cream to yellow brown [6,37] although there is no clear evidence about an association between a darker color of opacities and frequency of PEB [39].

It has been hypothesized that the influence of the severity of HSPM on the subsequent development of MIH could depend on the time at which a given etiological factor exerts its influence [10,25,37]. The calcification of SPM and FPM begins at around 18 weeks of gestation and at birth, respectively; subsequently, these two disorders have the same period of development. Mittal and Sharma [29] reported that the etiological insult acting on a late stage of mineralization of SPM produces milder HSPM because the mature dental germ is less susceptible, while MIH is more severe due to the immature stage of FPM mineralization. However, our findings and those of other studies on the relationship between HSPM and MIH do not corroborate this hypothesis, which should be further analyzed.

Our study encompasses various aspects that directly contribute to a better understanding of the relationship between two disorders, MIH and HSPM, which significantly impact a child’s oral health and psychophysical well-being. However, the primary objective of research in this field is to enhance preventive strategies and treatment options for this disease. Concerning the treatment of MIH, the European Academy of Paediatric Dentistry (EAPD) published a comprehensive policy document in 2021 [40], which provides an update to the “Best Clinical Practice guidance for clinicians dealing with children presenting with Molar-Incisor-Hypomineralisation (MIH)” released in 2010 [41]. In this document, the EAPD acknowledges that despite the increasing number of studies on the treatment of these disorders, the evidence remains limited and conventional restorative approaches continue to be the most commonly employed. As a result, the EAPD recommends the use of all available tools for the treatment of MIH teeth while emphasizing the importance of painless and effective treatment plans that prioritize the well-being of pediatric patients across dental, oral, medical, and social dimensions. In this regard, it is worth mentioning recent research that has shown promising results in this regard. For instance, the use of biomimetic zinc-hydroxyapatite has demonstrated positive outcomes in addressing hypersensitivity [42]. Additionally, resin infiltration has proven effective in the remineralization and masking of MIH white spots [43].

These innovative current and future treatment options emphasize the importance of exploring novel approaches to tackle the challenges presented by MIH and HSPM.

## 5. Limitations

Cross-sectional design: This study has a cross-sectional design. Longitudinal studies would provide stronger evidence.

Sample selection: Although the size of the MIH and non-MIH samples was large, with a total of 426 children included, the number of patients with a co-occurrence of both disorders was relatively small due to prevalence rates of MIH in our country.

The study was conducted in a specific location (Madrid, Spain), which may limit the general applicability of the findings to other populations or settings considering the range of prevalence of MIH and HSPM in different geographical areas.

## 6. Conclusions

Based on the results of this study, the following conclusions can be drawn:

HSPM can be considered a specific but not very sensitive predictor of MIH; the absence of HSPM in the primary dentition does not rule out the subsequent development of MIH.

Children affected by HSPM are more likely to develop MIH; when both disorders are associated, the severity of HSPM is related to the severity of subsequent MIH but not to the involvement of incisors by MIH.

In the context of MIH, taking proactive measures to address identifiable causes for preventive purposes is currently not feasible. Given the predictive nature of HSPM in relation to MIH, it is imperative that all healthcare practitioners, including pediatric dentists, hygienists, and pediatricians, actively engage in community surveillance programs focused on these children. This collaborative effort significantly enhances the efficacy of prevention and treatment strategies for enamel development disorders.

## Figures and Tables

**Table 1:** EAPD diagnostic criteria for MIH.

At least one FPM affected by the defect *
White-yellow-brown demarcated opacities
Post-eruptive enamel breakdown (PEB) associated with opacities
Extensive atypical caries with surrounding opacities or in low-risk surfaces
Atypical restorations; crowns if MIH is found in other teeth
Extractions due to MIH
Eruption failure of a molar or an incisor

* Lesions on the incisors are only diagnosed as MIH if at least one FPM is also affected.

**Table 2:** Inclusion/exclusion criteria in MIH and no-MIH samples.

To have all SPM preserved, unless one or more showed HSPM
Absence of other developmental defects (dentinogenesis imperfecta, amelogenesis imperfecta, fluorosis…) Absence of syndromes or craniofacial anomalies
Lack of consanguinity with other selected subjects
Availability of high quality digital intraoral photographs onto a 40-inch screen

**Table 3:** Gender, age, and age distribution of subjects with and without MIH.

	N	MIH (n = 213)	No MIH (n = 213)	*p* Value
Gender				0.845
Male	187	95 (44.6%)	92 (43.2%)	
Female	239	118 (54.4%)	121 (56.8%)	
Mean Age	426	8.43 ± 0.93	8.46 ± 0.92	0.335
Age distribution				0.876
7 years	67	35 (16%)	32 (15%)	0.790
8 years	168	82 (38.4%)	86 (40.4%)	0.766
9 years	131	68 (31.9%)	63 (29.6%)	0.674
10 years	60	28 (13.1%)	32 (15%)	0.676

Statistically significant *p* < 0.05.

**Table 4:** HSPM as a predictive test for MIH.

HSPM	MIH		Total
	No	Yes	
**No**	* TN = 189	FN = 134	323
	SP = 88.7%		
	NPV = 58.8%		
**Yes**	FP = 24	TP = 79	103
		SE = 37.1%	
		PPV = 76.7%	
**Total**	213	213	426

* TN true negative, FN false negative, NPV negative predictive value, SP specificity, FP false positive, TP true positive, PPV positive predictive value, SE sensitivity.

**Table 5:** Frequency of HSPM in patients with and without MIH.

	N	MIH (n = 213)	No MIH (n = 213)	*p* Value
**HSPM**				<0.001 *
Yes	103	79 (37.1%)	24 (11.3%)	
No	323	134 (62.9%)	189 (88.7%)	

* Statistically significant *p* < 0.05.

**Table 6:** Frequency of HSPM in patients with MIH regarding MIH severity.

	N	Mild MIH (n = 148)	Severe MIH (n = 65)	*p* Value
**HSPM**				0.296
Yes	79	51 (34.5%)	28 (43.1%)	
No	134	97 (65.5%)	37 (56.9%)	

Statistically significant *p* < 0.05.

**Table 7:** Relationship between MIH and HSPM severity in patients with co-occurrence of both disorders.

	N	Mild HSPM (n = 46)	Severe HSPM (n = 33)	*p* Value
**MIH severity**				<0.001 *
Mild	49	43 (87.8%)	6 (12.2%)	
Severe	30	3 (10%)	27 (90%)	

* Statistically significant *p* < 0.05.

**Table 8:** Relationship between severity of HSPM and involvement of only molars or molars + incisors by MIH in patients with co-occurrence of both disorders.

	N	Mild HSPM (n = 46)	Severe HSPM (n = 33)	*p* Value
**MIH**				0.956
Only molars	35	21 (60.0%)	14 (40.0%)	
Molars + incisors	44	25 (56.8%)	19 (43.2%)	

Statistically significant *p* < 0.05.

## Data Availability

The data presented in this study are available on request from the corresponding author. The data are not publicly available due to privacy/ethical reasons.
